# Effects of Curcumin Treatment in a Diabetic Neuropathic Pain Model of Rats: Involvement of c-Jun N-Terminal Kinase Located in the Astrocytes and Neurons of the Dorsal Root Ganglion

**DOI:** 10.1155/2021/8787231

**Published:** 2021-01-19

**Authors:** Hanwool Park, Jong-Hyuk Lee, Ji Hoon Sim, Jihoon Park, Seong-Soo Choi, Jeong Gil Leem

**Affiliations:** Department of Anesthesiology and Pain Medicine, University of Ulsan College of Medicine, Asan Medical Center, Seoul, Republic of Korea

## Abstract

Curcumin (diferuloylmethane) is a major component of turmeric, which is isolated from the rhizomes of *Curcuma longa* L. from the family Zingiberaceae. It is used as a dietary pigment for curry and in traditional Indian medicine for its anti-inflammatory and attenuating pain effects. This study aimed to evaluate the beneficial effects of curcumin in a rat model of diabetic neuropathic pain. Additionally, we investigated the involvement of the phosphorylated form of c-Jun N-terminal kinase (pJNK) located in the neurons and astrocytes of the dorsal root ganglion (DRG). To induce diabetic neuropathic pain in rats, 50 mg/kg of streptozotocin (STZ) was intraperitoneally injected. After 4 weeks, rats were administered the vehicle, 10 mg/kg/day curcumin, or 50 mg/kg/day curcumin orally for 4 consecutive weeks. One day after the final drug administration, we performed behavioral tests to measure responses of rats to mechanical, heat, cold, and acetone-induced cold stimuli. After behavioral tests, pJNK expression in the DRG was evaluated using western blot assay and immunohistochemistry. Curcumin treatment for 4 consecutive weeks in STZ-induced diabetic neuropathic pain rats improved behavioral responses to mechanical, cold, and thermal stimuli. Increased pJNK expression in the astrocytes and neurons of the DRG in STZ-induced diabetic neuropathic pain rats was reduced by curcumin treatment for 4 consecutive weeks. We suggest that curcumin can be an option for the treatment of diabetes-related neuropathic pain, and one of the mechanisms that underlie the action of curcumin may involve pJNK expression in the astrocytes and neurons of the DRG.

## 1. Introduction

Neuropathic pain is one of the most common complications of diabetes mellitus (DM) and an estimated one-third of patients with DM have painful diabetic neuropathy [1]. It is characterized by hyperalgesia (increased sensitivity to pain), allodynia (pain sensation to nonpainful stimulation), dysesthesia (an unpleasant sense of touch), and paresthesia (abnormal sensation without a cause) and is caused by either peripheral nerve damage or changed neuronal signaling. However, molecular and cellular mechanisms that underlie neuropathic pain are not fully understood.

Curcumin (diferuloylmethane) is a natural product from the *Curcuma longa* L. from the family Zingiberaceae. It is a member of the curcuminoid family and used as a dietary pigment in curry and an ancient medicine for many purposes including asthma, allergy, anorexia, coryza, cough, hepatic disease, and sinusitis. Nowadays, curcumin is known to play an important role in inflammatory conditions. Curcumin decreases postsurgical allodynia [2], mitigates diabetic neuropathic pain [3], prevents the development of diabetic neuropathy [4], and decreases pain associated with depression [5]. Currently, the main analgesic mechanism of curcumin is thought to involve signals such as tumor necrosis factor-*α* (TNF-*α*), nitric oxide (NO) [6], and extracellular signal-regulated kinase (ERK) [7] in the anti-inflammatory pathway. Also, there are other analgesic mechanisms involved in curcumin such as activation of NF-*κ*B [8], noradrenergic and serotoninergic system *β*2-adrenoreceptor and 5-HT1A receptor [9], NO-cGMP-ATP-sensitive *K*+ channel pathways [10], TTX-R sodium currents [11], and opioid system [12].

Similar to ERK, c-Jun N-terminal kinase (JNK) is a member of the mitogen-activated protein kinase (MAPK) family. It regulates neuronal functions, immunological actions, and embryonic development [13]. JNK is also activated in the neurons of the dorsal root ganglion (DRG) and spinal cord after the ligation of a spinal nerve resulting in neuropathic pain [14]. JNK is activated when it becomes phosphorylated (phosphorylated JNK (pJNK)) [13]. Interestingly, JNK is also one of the most important kinases implicated in hyperglycemia and the pathogenesis of diabetes [15].

Curcumin is known to inhibit JNK activation [16–18]. However, it is unknown whether curcumin affects JNK in the neurons and astrocytes of the DRG. DRG is important for communication between the peripheral and the central nervous systems, and it is a target for pain management [19]. Therefore, the present study investigated the effects of curcumin on diabetic neuropathic pain induced by streptozotocin (STZ) in rats and on pJNK expression in the astrocytes and neurons of the DRG.

## 2. Materials and Methods

This study was approved by the Institutional Animal Care and Use Committee of the Asan Institute for Life Sciences, Asan Medical Center (approval number 2012-13-211). The committee follows the guidelines of the Institute of Laboratory Animal Resources. We performed all experiments according to the ethical guidelines of the International Association for the Study of Pain [20]. The animals were euthanized painlessly after the completion of all the experiments.

### 2.1. Experimental Animals

Male Sprague-Dawley rats (200∼250 g) were housed in a standard cage under a 12-hour light-dark cycle. Humidity and temperature were controlled at 65 ± 5% and 21 ± 1°C, respectively. Water and food were freely provided. The animals were allowed to habituate to the laboratory environment for at least 2 hours before performing behavioral experiments.

### 2.2. Generation of the Diabetic Neuropathic Pain Model and the Experiment Protocol

Diabetic neuropathic pain can be easily induced by the systemic administration of STZ [21–24]. Usually, rats can manifest diabetic neuropathy 4 weeks after a single administration of STZ [22, 25].

**Figure 1:**
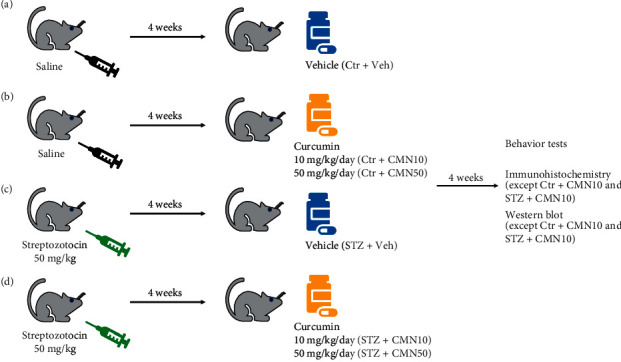
Simple experimental scheme. (a, b) Groups of rats were injected with saline solution at 0.9% intraperitoneally (IP). Four weeks later, they received either vehicle (Ctr + Veh), 10 mg/kg/day (Ctr + CMN10), or 50 mg/kg/day of curcumin (Ctr + CMN50) orally for 4 consecutive weeks. The experiments were subsequently conducted. (c, d) Groups of rats were injected with 50 mg/kg of streptozotocin (STZ) IP. Four weeks later, they received vehicle (STZ + Veh), curcumin 10 mg/kg/day (STZ + CMN10), or 50 mg/kg/day (STZ + CMN50) orally for 4 weeks before the start of the experiments.

As shown in the graphical abstract (Figure 1), rats were randomly assigned to six groups (*n* = 10 per group): (1) control (Ctr) + vehicle (Veh), (2) Ctr + 10 mg/kg/day of curcumin (Ctr + CMN10), (3) Ctr + 50 mg/kg/day of curcumin (Ctr + CMN50), (4) STZ + Veh, (5) STZ + CMN10, and (6) STZ + CMN50. Initially, saline solution at 0.9% (Ctr) or 50 mg/kg of STZ was injected intraperitoneally (IP). After 4 weeks, rats were also orally treated with 10 mg/kg/day curcumin, dissolved in 5% alcohol and 5% dextrose in water (5DW), and either 50 mg/kg/day CMN or vehicle (Veh, 5DW) for 4 consecutive weeks. One day after the final drug administration, we performed behavioral tests to measure the responses of rats to mechanical, heat, cold, and acetone-induced cold stimuli. After behavioral tests, we performed western blot assay and immunohistochemistry for pJNK in the DRG. The Ctr + CMN10 and STZ + CMN10 groups were excluded from western blot analysis and immunohistochemistry because the CMN50 groups showed better behavioral effects of curcumin than the CMN10 groups.

### 2.3. Curcumin and Antibodies

We used curcumin (Santacruz, Dallas, Texas, USA, #sc-200509) in this study. Glial fibrillary acidic protein (GFAP, Cell Signaling Technology, Danvers, USA, #3670, 1 : 100), pJNK (Cell Signaling Technology, Danvers, USA, #5668, 1 : 50 for IHC, 1 : 1000 for western blot), Neurofilament 200 (NF200, Sigma-Aldrich, St. Louis, Missouri, USA, N5389, 1 : 40), and *β*-actin (BETHYL, Montgomery, Texas, USA, A300-491, 1 : 10000) were used as primary antibodies. BETHYL A120-101D3, A90-116D2, and A120-101P were used as secondary antibodies.

### 2.4. Behavioral Tests

Behavioral tests were performed to measure responses to mechanical, heat, cold, and acetone-induced cold stimuli. All experiments were performed during the light phase (10 : 00–16 : 00).

Mechanical allodynia was tested using a Von Frey filament (Stoelting, Wood Dale, IL). The plantar surface of the hindfoot was tested. We used eight types of calibrated von Frey filaments (0.41 g, 0.70 g, 1.20 g, 2.00 g, 3.63 g, 5.50 g, 8.50 g, and 15.10 g). We pressed the plantar surface of the feet for 6 seconds with these filaments. Rapid withdrawal or startling was measured as a positive response. The 50% withdrawal threshold was measured by using the up-down method [26].

Cold allodynia against chemical stimuli was tested using the acetone [27]. A drop of acetone was applied to the midplantar surface of the hind paw for five times. The frequency of the paw withdrawals in response to the acetone was measured.

Allodynia and hypersensitivity to thermal and cold stimuli were analyzed using a hot/cold plate apparatus (Ugo Basile, Comerico, Italy). For thermal allodynia and hypersensitivity testing, the hot plate temperature was adjusted to 42 ± 0.1°C and 50 ± 0.1°C, respectively [28]. For cold allodynia and hypersensitivity testing, the cold plate temperature was adjusted to 10 ± 0.1°C and 4 ± 0.1°C, respectively [28]. The latency to first hind paw withdrawal or licking was measured. Every rat was tested three times. The cut-off time for allodynia was 30 seconds for the thermal test and 100 seconds for the cold test [29]. The cut-off time for hypersensitivity was 15 seconds for both thermal and cold tests [28].

### 2.5. Western Blot

After the final behavioral test, DRG tissues were isolated for western blot analysis. Tissues were washed twice with cold Tris-buffered saline and lysed with 2% SDS lysis buffer containing 0.1 mM Na_3_VO_4_, 3 mg/mL aprotinin, and 20 mM NaF. Because the DRG of a single rat was not sufficient for western blotting, we mixed samples from six rats for each group. After sonication, the protein concentration was measured using a detergent compatible protein assay reagent (Bio-Rad Laboratories, Hercules, California, USA). The total protein samples (40 *μ*g) were used for the blotting. After immunoblotting with the antibodies, the ECL-plus solution (Amersham Pharmacia Bioscience, Piscataway, New Jersey, USA) was used. The specific blotted signals for pJNK and *β*-actin were quantified using the ImageJ freeware (NIH, Betheseda, Maryland, USA).

### 2.6. Immunohistochemistry

After the completion of behavioral tests, we injected rats with zoletil (60 mg/kg) and xylazine (10 mg/kg) intraperitoneally to enable DRG sampling for immunostaining. We perfused rats with 4% paraformaldehyde via the left ventricle of the heart for the fixation. The DRG was isolated and fixed immediately in 4% paraformaldehyde. The fixed DRG samples were embedded in paraffin. We sectioned the DRG samples into 10 *μ*m thick sections using a microtome and mounted the sections onto slides. Deparaffinization was performed using xylene and ethanol. The blocking solution comprised 5% donkey serum, 0.3% Triton X-100, and 1% bovine serum albumin in phosphate-buffered saline. After incubating overnight with GFAP, NF200, and pJNK antibodies, the tissues were incubated with secondary antibodies for 2 hours. We acquired images using a confocal microscope (LSM780, Carl Zeiss, Obercochen, Germany) and used Image pro plus ver 5.1 imaging software (Media Cybernetics, Rockville, Maryland, USA) for analysis.

### 2.7. Statistical Analysis

Data are presented as means±standard error. Behavioral test data were evaluated using one-way analysis of variance (ANOVA) with Bonferroni post hoc test using GraphPad Prism Version 5.01 for Windows (GraphPad Software, San Diego, CA, USA). *p* value <0.05 was considered as significant.

## 3. Results

### 3.1. Effects of Curcumin on STZ-Induced Diabetic Neuropathic Painful Responses in Rats: Behavioral Responses to Mechanical and Thermal Stimuli

**Figure 2:**
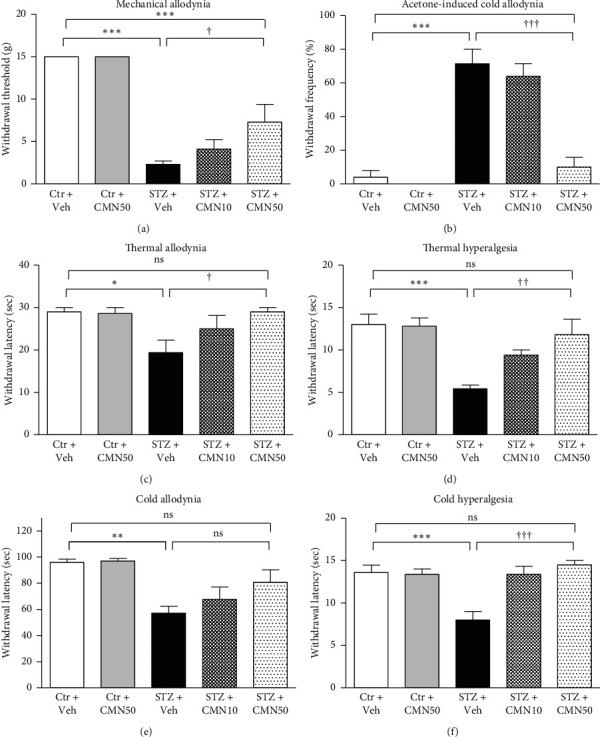
Effects of curcumin (CMN) administered orally on the diabetic neuropathic painful behavior induced by streptozotocin (STZ). STZ (50 mg/kg) was intraperitoneally injected to induce diabetic neuropathy in rats. After 4 weeks, rats were treated orally with vehicle and either 10 mg/kg/day or 50 mg/kg/day of CMN for 4 consecutive weeks. One day after final drug administration, behavioral tests were performed to measure responses to mechanical (a), acetone-induced cold (b), thermal ((c) 42°C and (d) 50°C), and cold ((e) 10°C and (f) 4°C) stimuli (*n* = 10 animals per group, one-way ANOVA with Bonferroni post hoc). ^*∗*^*p* < 0.05, ^*∗∗*^*p* < 0.001, and ^*∗∗∗*^*p* < 0.001 compared to the Ctr + Veh group. ^†^*p* < 0.05, ^††^*p* < 0.01, and ^†††^*p* < 0.001 compared to the STZ + Veh group.

We subjected the STZ-induced diabetic neuropathic pain rats to a battery of allodynia and hyperalgesia tests (Figure 2). STZ-induced diabetic neuropathic pain rats showed significantly reduced paw withdrawal threshold to mechanical stimuli. Consecutive treatment of diabetic neuropathic pain rats with curcumin for 4 weeks increased the withdrawal threshold to mechanical stimuli (*p* < 0.05) although the threshold was not fully recovered to the level of the control group (Figure 2(a)). STZ-induced diabetic neuropathic pain rats also exhibited increased frequency of paw withdrawal in response to acetone-induced cold stimuli. This increased frequency of the paw withdrawals induced by acetone in STZ-induced diabetic neuropathic pain rats significantly reduced following consecutive treatment with curcumin for 4 weeks (Figure 2(b), *p* < 0.001). As shown in Figures 2(c) and 2(d), paw withdrawal latency to thermal stimuli (42°C and 50°C) also significantly decreased in STZ-induced diabetic neuropathic pain rats. Consecutive treatment of diabetic neuropathic pain rats with curcumin for 4 weeks significantly reversed the decreased paw withdrawal latency to thermal stimuli (thermal allodynia, Figure 2(c), *p* < 0.05; and thermal hyperalgesia, Figure 2(d), *p* < 0.01). Similarly, paw withdrawal latencies to cold stimuli (10°C and 4°C) significantly decreased in STZ-induced diabetic neuropathic pain rats (Figure 2(e), *p* < 0.01; Figure 2(f), *p* < 0.001). Consecutive treatment of diabetic neuropathic pain rats with curcumin for 4 weeks reversed the decreased paw withdrawal latency to 10°C (Figure 2(e), *p* > 0.05) and 4°C cold stimuli (Figure 2(f), *p* < 0.001). Although the reversal of paw withdrawal latency to 10°C was not significant (Figure 2(e)), the tendency was prominent.

### 3.2. Effects of Curcumin on pJNK Expression in STZ-Induced Diabetic Neuropathic Pain Rats: Involvement of pJNK in the Astrocytes and Neurons of the DRG

**Figure 3:**
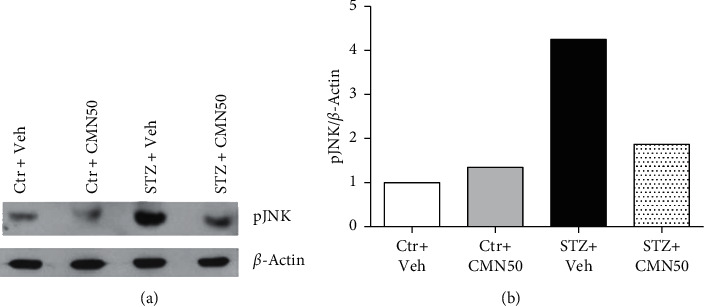
Western blot analysis of pJNK protein expression in the dorsal root ganglion (DRG). Total protein extraction from DRG was used for the western blot analysis. pJNK expression was increased in streptozotocin- (STZ-) induced diabetic neuropathic pain rats (STZ + Veh, *n* = 6), which was subsequently normalized by treatment with 50 mg/kg/day of curcumin (CMN) for 4 consecutive weeks (STZ + CMN50, *n* = 6). *ß*-Actin is a loading control.

Immunoblot analyses of the DRG revealed that the levels of pJNK protein expression were elevated in STZ-induced diabetic neuropathic pain rats (Figure 3). Consecutive treatment with 50 mg/kg curcumin for 4 consecutive weeks decreased the elevated pJNK expression induced by STZ administration. The treatment of sham control groups with curcumin did not change the amount of pJNK protein expressed.

**Figure 4:**
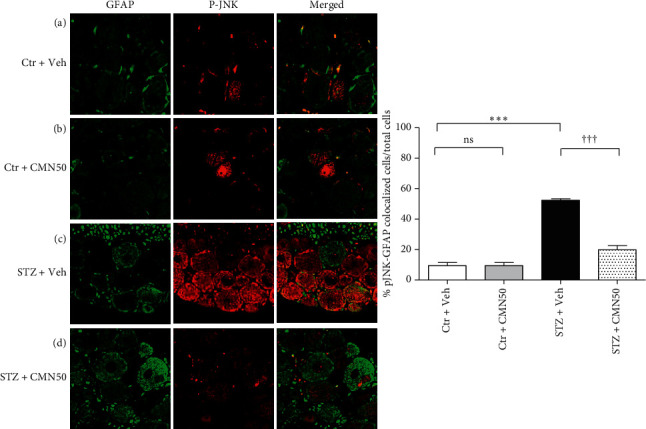
Immunohistochemistry for pJNK (red, pJNK antibody) expression in the astrocytes of the dorsal root ganglion (DRG) (green, GFAP antibody). Treatment of streptozotocin- (STZ-) induced diabetic neuropathic pain rats (STZ + CMN50) with 50 mg/kg/day of curcumin (CMN) for 4 consecutive weeks normalized pJNK expression in the astrocyte of the DRG. (a) Control group (Ctr + Veh, *n* = 4); (b) curcumin-treated control group (Ctr + CMN50, *n* = 4); (c) STZ-induced diabetic neuropathic pain rats (STZ + Veh, *n* = 4); (d) curcumin-treated STZ-induced diabetic neuropathic pain rats (STZ + CMN50, *n* = 4). ^*∗∗∗*^*p* < 0.001 compared to the Ctr + Veh group; ^†††^*p* < 0.001 compared to the STZ + Veh group.

**Figure 5:**
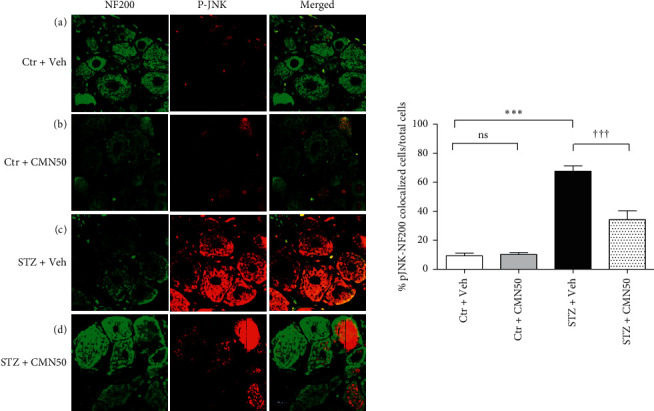
Immunohistochemistry for pJNK (red, pJNK antibody) expression in the neurons of the dorsal root ganglion (DRG) (green, NF200 antibody). Treatment of streptozotocin- (STZ-) induced diabetic neuropathic pain rats (STZ + CMN50) with 50 mg/kg/day of curcumin (CMN) for 4 consecutive weeks normalized pJNK expression in the neurons of the DRG. (a) Control group (Ctr + Veh, *n* = 4), (b) curcumin-treated control group (Ctr + CMN50, *n* = 4), (c) STZ-induced diabetic neuropathic pain rats (STZ + Veh, *n* = 4), and (d) curcumin-treated STZ-induced diabetic neuropathic pain rats (STZ + CMN50, *n* = 4). ^*∗∗∗*^*p* < 0.001 compared to the Ctr + Veh group; ^†††^*p* < 0.001 compared to the STZ + Veh group.

Immunohistochemical analysis revealed an increase in pJNK immunoreactivity of DRG cells. These cells colocalized with GFAP (astrocyte marker) and NF200 (neuronal marker) positive cells, as shown in Figures 4(c) and 5(c), respectively. Consecutive treatment of STZ-induced diabetic neuropathic pain rats with 50 mg/kg curcumin for 4 weeks decreased the number of pJNK immunoreactive cells that were colocalized with both GFAP and NF200 in the DRG (Figures 4(d) and 5(d), respectively). Vehicle (control) or curcumin administration to the control group did not alter the pJNK immunoreactivity of the DRG cells (Figures 4(a)and 4(b) and 5(a)and 5(b), respectively).

## 4. Discussion

We examined the ability of curcumin to ameliorate neuropathic pain behaviors in STZ-induced diabetic neuropathic pain rats. Curcumin treatment decreased mechanical, cold, and thermal allodynia and hyperalgesia. Consistent with the findings of this study, previous studies also showed similar effects of curcumin on neuropathic pain. The treatment of STZ-induced diabetic neuropathic pain animals with curcumin reduced paw licking and tail-withdrawal response [3], decreased mechanical allodynia [12], improved sensorimotor deficits [12], and decreased thermal and mechanical hyperalgesia [30]. In another rat model of neuropathic pain from brachial plexus avulsion, administration of curcumin markedly increased mechanical withdrawal threshold and decreased paw-withdrawal frequency to cold stimuli [31]. Additionally, two clinical trials involving curcumin confirmed its beneficial effects on diabetic neuropathy [32, 33]. Parallel, double-blind randomized, placebo-controlled trials showed that the treatment of patients with type 2 DM complicated by peripheral neuropathy with nanocurcumin supplements reduced glycated hemoglobin (HbA1c), fasting blood sugar, total neuropathy score, total reflex score, temperature, depression, and anxiety scores [32, 33].

The mechanism of action of curcumin effect in diabetic neuropathy is currently under investigation. The pathogenesis of neuropathic pain is complex, making it difficult to identify the mechanism underlying the effect of curcumin on neuropathy. Diabetic neuropathy is thought to occur as a result of hyperglycemia and lack of neurotrophic support from insulin/C-peptide [34]. Hyperglycemia causes cytokine releases, oxidative stress, lipid peroxidation, and neuroinflammation [35, 36]. Curcumin exhibited anti-inflammatory effects through the inhibition of TNF-*α*, NO, ERK, and nuclear factor-kappa B [6, 7, 34, 37]. Liu and coworkers have also showed that curcumin can decrease neuropathic pain by downregulation of spinal interleukin-1*β* via suppressing NAcht leucine-rich-repeat protein 1 inflammasome, and activation of Janus kinase 2-signal transducer and activator of transcription 3 cascade [38]. Furthermore, it has been reported that curcumin downregulates the activation of the JNK pathway in hyperglycemia models *in vitro* and *in vivo*. The *in vitro* model was induced by applying bisphenol A (BPA) into LO2 cells [16], while the *in vivo* model was induced using STZ followed by the examination of the spinal cord [18]. In western blot analysis of the DRG in the present study, curcumin treatment normalized the increased DRG pJNK expression induced by STZ administration. Because the DRG of a single rat was not sufficient for western blotting, we mixed samples from six rats for each group. Although we did not perform statistical analysis on the western blot results, the tendency toward a normalized pJNK expression might be enough to suggest that the analgesic effect of curcumin involves DRG pJNK protein expression in STZ-induced diabetic neuropathic pain rats.

In STZ-induced diabetic neuropathic pain models, pJNK is induced in the spinal cord neurons but not in the astrocytes [39]. However, they did not investigate which cell type located in the DRG expresses JNK although the immunoblot showed an increase in JNK levels of DRG in STZ-induced diabetic neuropathic rats. JNK is also activated in the spinal astrocytes in other chronic pain conditions, including nerve injury induced by sciatic nerve ligation [14], inflammation induced by complete Freund's adjuvant [40], bone cancer [41], and skin cancer [42]. Additionally, JNK phosphorylation increased in the neurons of the DRG in an STZ-induced diabetic neuropathic pain animal model [43]. However, there has been no study on pJNK expression in the astrocytes in an STZ-induced diabetic neuropathic pain model. In the immunohistochemical analysis to determine DRG cells that expressed pJNK, we found that pJNK was activated not only in the neurons but also in the astrocytes of the DRG in STZ-induced diabetic neuropathic pain rats. The present study demonstrated that curcumin normalized pJNK expression in the astrocytes and neurons of the DRG in STZ-induced diabetic neuropathic pain rats. This suggests that curcumin administration decreases diabetic neuropathic pain responses by normalizing functional JNK activation in DRG cells in STZ-induced diabetic neuropathic pain rats. Additionally, the loss of A*δ* fibers leads to cold hyperalgesia, while the loss of C fibers leads to decreased thermal and mechanical pain thresholds [44]. Curcumin may differentially affect the aforementioned fibers. This may explain why only mechanical allodynia was not fully improved. However, our immunohistochemical analysis could not differentiate between the effect of curcumin on A*δ* fibers and that on C fibers.

## 5. Conclusions

We demonstrated the effect of curcumin in a rat model of diabetic pain induced by STZ. Curcumin may be useful in the management of diabetic neuropathic pain characterized by hyperalgesia or allodynia. In addition, we found that pJNK expression in the astrocytes and neurons of the DRG was increased by STZ-induced diabetic neuropathic rats. This increased pJNK expression might be decreased by curcumin treatment. We suggest that curcumin can be an option for the treatment of diabetes-related neuropathic pain and that one of the mechanisms of action of curcumin may involve pJNK expression in the astrocytes and neurons of the DRG.

## Data Availability

The original data used to support the findings of this study are available from the corresponding author upon request.
